# Anomalies and Curiosities of Medical Literature

**Published:** 1915-06-05

**Authors:** 


### THE EDITOR'S LETTER-BOX.

### Anomalies and Curiosities of Medical Literature.

*To the Editor of* The Hospital.

Sir,—"Verify your references" is a very old maxim. The following instance has only recently come to light (see *Boston Med. and Surg. Jour.*, April 22, 1915). One read long ago in that journal of a case of eight children at a birth; the note is as follows: "On August 21, Mrs. Timothy Bradlee, of Trumbell County, Ohio, gave birth to eight children, three girls and five boys. They are all living and are healthy, but quite small. Mr. Bradlee was married six years ago to Eunice Mowery, who weighed 273 pounds on the day of her marriage. She has given birth to two pairs of twins, and now eight more, making twelve children in six years. Mrs. Bradlee was a triplet, her mother and father being twins, and her grandmother the mother of five pairs of twins."

The sequel is as follows: A gentleman (a Mr. G. H. Parker) of the Zoological Laboratory, Harvard University, apparently working at the subject, had occasion to question the reference, and in 1914 wrote to a Mr. Tayler, Clerk of the Courts, Trumbell County. The reply he received is published in the same medical journal that first reported the case. In the course of the reply, dated March 30, 1914, the Clerk wrote: "In regard to the item in the medical journal, I would say that, after inquiry, I am informed that there is no truth in the statement. It seems that a practical joker of those days went into one of the newspaper offices here and set up an article which he succeeded in having printed in one or two copies of the paper, and then took the article out and distributed the type in their proper places. Securing the copies which had the article in, sent the same to a New York paper, thinking he had accomplished a great joke. This is all the information I can obtain in regard to the matter, but can state that there is no truth or foundation in the report whatever."

These unverified references cause untold trouble, and in this case the statement has been incorporated with other similar cases in that valuable book of reference, Gould and Pyle's "Anomalies and Curiosities of Medicine," 1897, p. 153, and into such special papers as that of Wilder on "Duplicate Twins," *American Journal of Anatomy*, vol. iii., p. 393, 1904.

It is particularly satisfactory that the misstatement is corrected at the earliest opportunity by the medical journal in which it occurred, though as many persons may have relied upon the record during the forty-three years that have elapsed since it was first published, the incident certainly points to the necessity of accurately checking unusual reports before publishing them as scientific literature.—Yours faithfully,

Medical Librarian.

